# Supplementary material for: Molecular Probes to Evaluate the Synthesis and Production Potential of an Odorous Compound (2-methylisoborneol) in Cyanobacteria
Source: Int J Environ Res Public Health. 2020 Mar 16;17(6):1933. doi: 10.3390/ijerph17061933 (PMC7142697; doi:10.3390/ijerph17061933)

Supplementary Figure S1. Location of the sampling site (red circle) in the estuary region of the Gong-ji stream

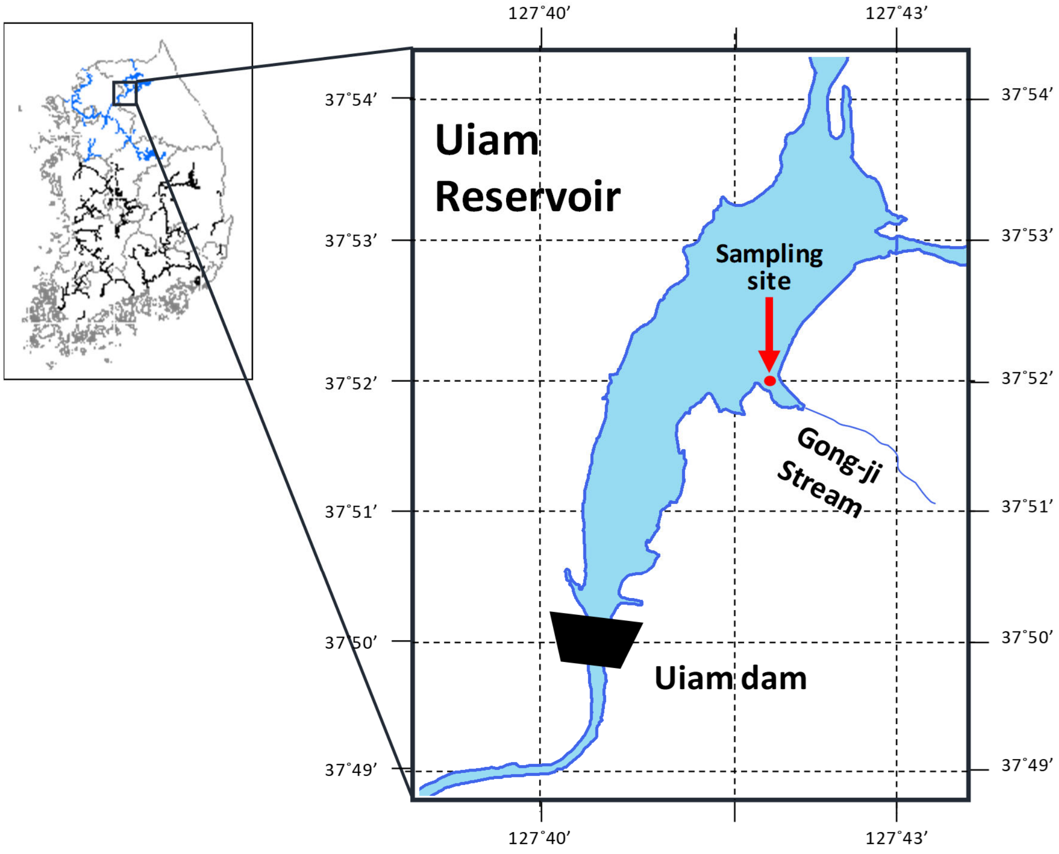

Supplementary Figure S2. Nonspecific band occurrence for all tested strains according to annealing temperature. The y-axis indicates the number of nonspecific DNA bands. Among the eight sets of primers, mibC132, mibC196, and mibC300 produced only one amplicon, and the amplicon had the expected size. No value is shown for the mibC127 primer, which did not produce amplicons.

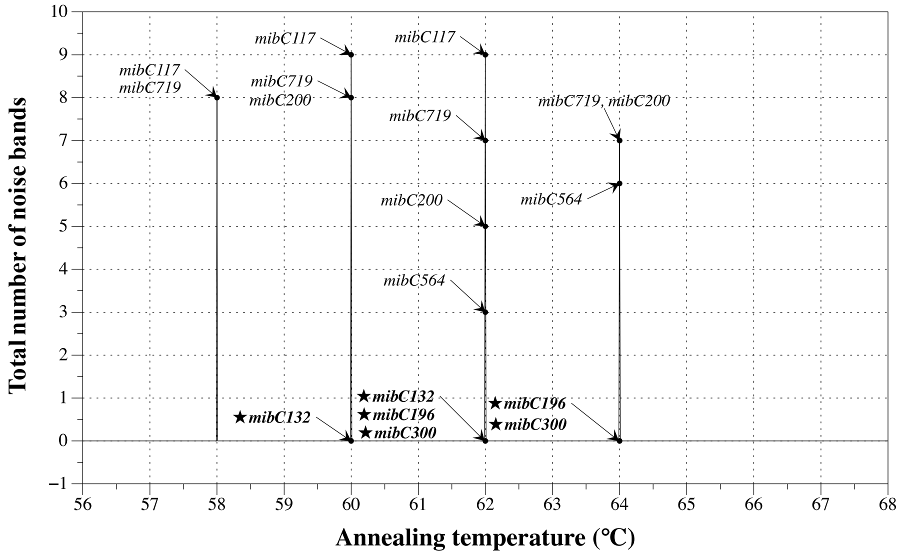

Supplementary Figure S3. Detection of *mibC* genes from the field samples by PCR. The presence of *mibC* genes in field samples collected from Gong-ji Stream sediment during January (a) and February (b) in 2016 was investigated by PCR using the primers developed in this study. Lane 1 shows a 100-bp DNA ladder, and the PCR products from mibC300 (1), mibC196 (2), and mibC132 (3) are shown at the expected sizes. Each relative quantification (RQ) value was calculated using Image J software (NIH, USA).

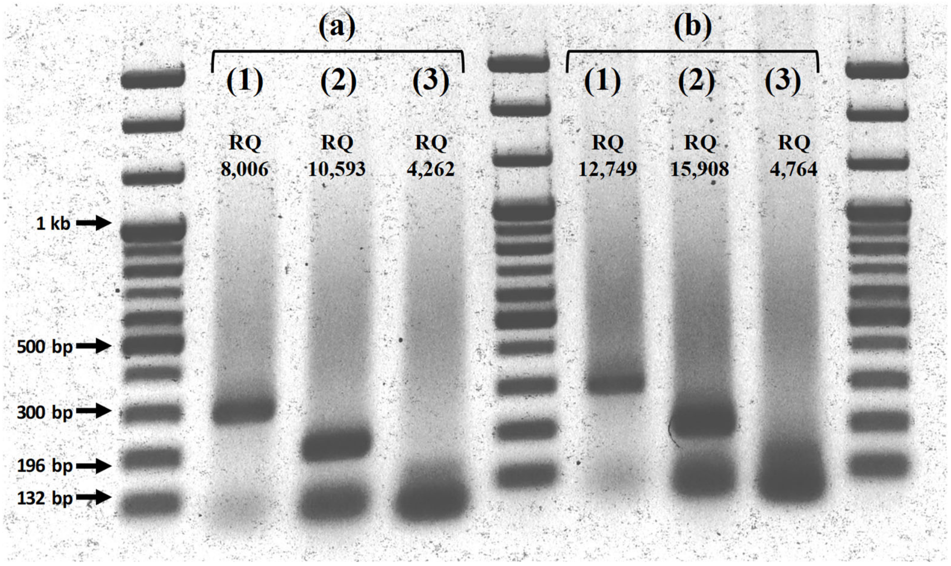

Supplementary Figure S4. Phylogenic analysis of 2-MIB-synthesizing gene (*mibC*) sequences obtained using the designed primers with sequences from the GenBank database. The DNA sequence of amplicons generated by each primer from the field samples was subjected to phylogenetic analysis. Phylogenic tree constructed with the gene sequences amplified using the primers (a) mibC132, (b) mibC196, and (c) mibC300. Phylogenic analysis of aligned sequences was performed using the maximum-likelihood method and did not indicate bootstrap values under bootstrap 50 (1,000 bootstrap replicates). Nostoc sp. UK1 strain was the root species.

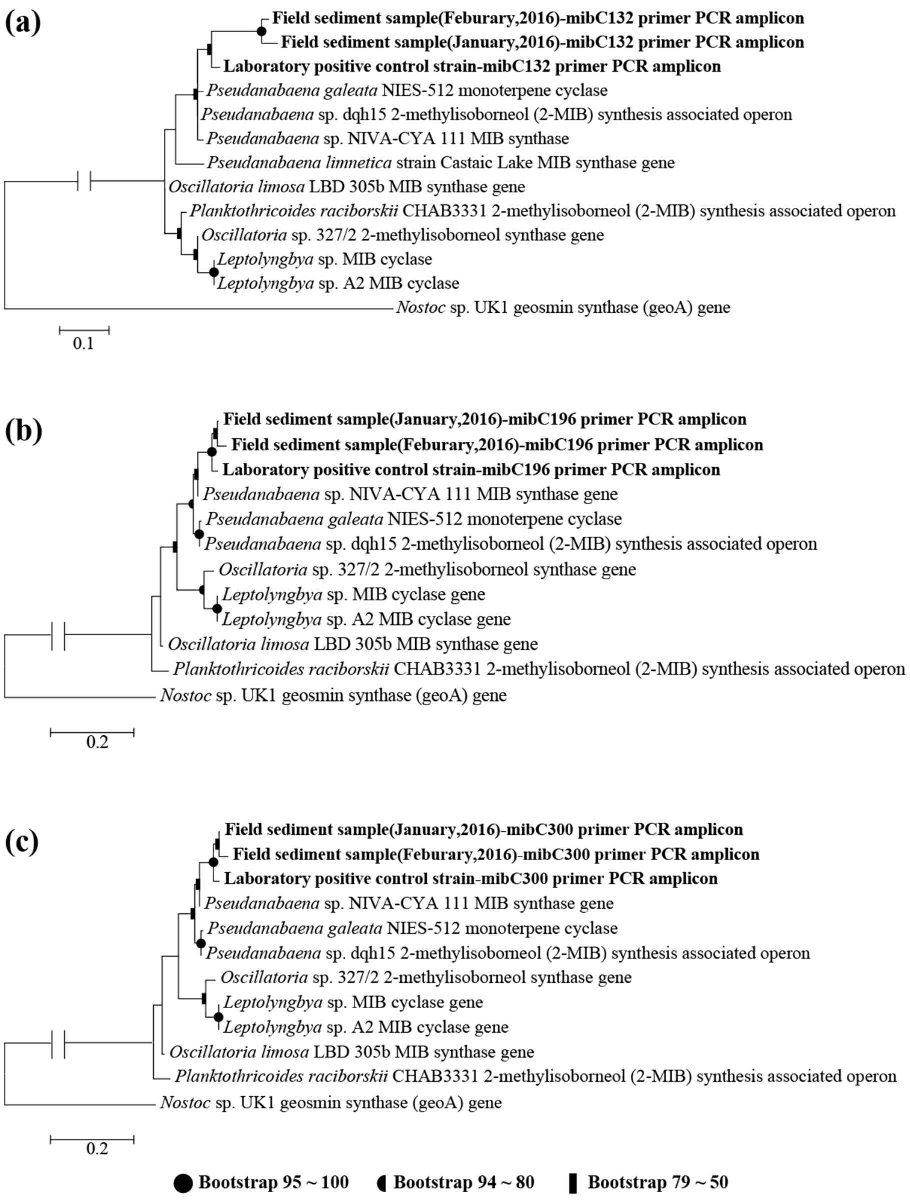

Supplementary Figure S5. Seasonal variation in *mibC* copy number (a), gene expression (b) in the water column and sediment, 2-MIB concentration (c), and *Pseudanabaena limnetica* cell density (d) in the water column of the Gong-ji Stream between February 2015 and February 2016.

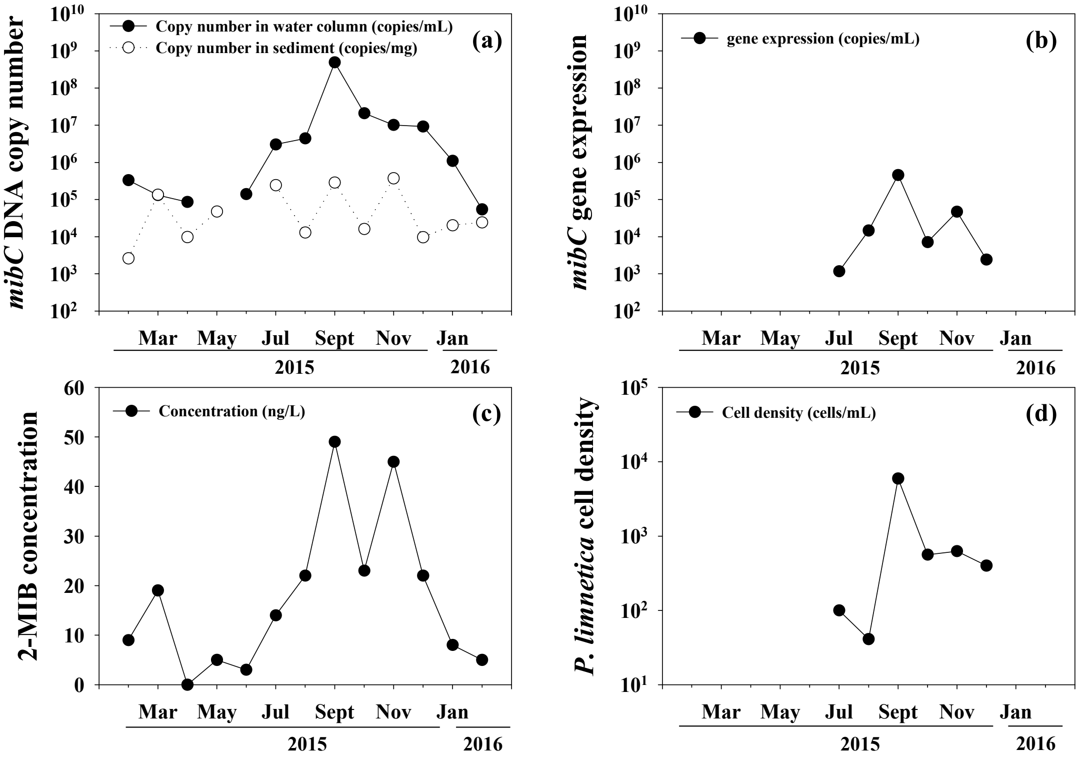

Supplement: Supplementary file 1 [file ijerph-17-01933-s001.pdf]
